# New Insights on Taxonomy, Phylogeny and Population Genetics of *Leishmania (Viannia)* Parasites Based on Multilocus Sequence Analysis

**DOI:** 10.1371/journal.pntd.0001888

**Published:** 2012-11-01

**Authors:** Mariana C. Boité, Isabel L. Mauricio, Michael A. Miles, Elisa Cupolillo

**Affiliations:** 1 Laboratório de Pesquisa em Leishmaniose, Fundação Oswaldo Cruz, Instituto Oswaldo Cruz, Rio de Janeiro, Brazil; 2 Department of Pathogen Molecular Biology, Faculty of Infectious and Tropical Diseases, London School of Hygiene and Tropical Medicine, London, United Kingdom; 3 Instituto de Higiene e Medicina Tropical/Unidade de Parasitologia e Microbiologia Médicas, Lisboa, Portugal; Yale School of Public Health, United States of America

## Abstract

The *Leishmania* genus comprises up to 35 species, some with status still under discussion. The multilocus sequence typing (MLST)—extensively used for bacteria—has been proposed for pathogenic trypanosomatids. For *Leishmania*, however, a detailed analysis and revision on the taxonomy is still required. We have partially sequenced four housekeeping genes—glucose-6-phosphate dehydrogenase (G6PD), 6-phosphogluconate dehydrogenase (6PGD), mannose phosphate isomerase (MPI) and isocitrate dehydrogenase (ICD)—from 96 *Leishmania (Viannia)* strains and assessed their discriminatory typing capacity. The fragments had different degrees of diversity, and are thus suitable to be used in combination for intra- and inter-specific inferences. Species-specific single nucleotide polymorphisms were detected, but not for all species; ambiguous sites indicating heterozygosis were observed, as well as the putative homozygous donor. A large number of haplotypes were detected for each marker; for 6PGD a possible ancestral allele for *L. (Viannia)* was found. Maximum parsimony-based haplotype networks were built. Strains of different species, as identified by multilocus enzyme electrophoresis (MLEE), formed separated clusters in each network, with exceptions. NeighborNet of concatenated sequences confirmed species-specific clusters, suggesting recombination occurring in *L. braziliensis* and *L. guyanensis*. Phylogenetic analysis indicates *L. lainsoni* and *L. naiffi* as the most divergent species and does not support *L. shawi* as a distinct species, placing it in the *L. guyanensis* cluster. BURST analysis resulted in six clonal complexes (CC), corresponding to distinct species. The *L. braziliensis* strains evaluated correspond to one widely geographically distributed CC and another restricted to one endemic area. This study demonstrates the value of systematic multilocus sequence analysis (MLSA) for determining intra- and inter-species relationships and presents an approach to validate the species status of some entities. Furthermore, it contributes to the phylogeny of *L. (Viannia)* and might be helpful for epidemiological and population genetics analysis based on haplotype/diplotype determinations and inferences.

## Author Summary

*Leishmania* is a protozoan genus comprising many species, some associated with a human neglected disease called leishmaniasis. This parasite is found worldwide and is transmitted by sand flies, having numerous domestic and sylvatic animals as reservoirs. *Leishmania* is genetically and ecologically diverse and it has been argued that this has an impact on the epidemiology of the disease. Many typing methods have been proposed for the study of this diversity, although a generally agreed methodology is still required. Also, there is still a lack of consensus on the validity of some species. Multilocus sequence typing (MLST) is a method for studying the population structure and diversity of pathogens, but before an MLST scheme can be proposed it is essential to undertake a detailed analysis and selection of the sequences that are to be included in the system. Here, we sequenced four gene fragments of 96 *L.* (*Viannia*) strains, representing most species from this subgenus. Our results showed a good agreement between the current species assignment and the multilocus sequence analysis. Evidence of genetic recombination was found and the phylogenetic relationships were determined. Overall the results point to the feasibility of an MLST scheme for *Leishmania* and indicate that the four gene fragments analyzed could form part of this typing system. This will certainly be a valuable approach for taxonomy, population genetics, and epidemiological studies of this pathogen.

## Introduction

*Leishmania* are the causative agents of leishmaniasis, which can present in different forms, from simple cutaneous to the deadly visceral disease, and are found in most tropical and sub-tropical regions. In spite of their morphological homogeneity, more than 20 species have been described for the *Leishmania* genus. Phenotypic diversity is observed not only between species, but also even in virulence levels among clones [1] which, upon interaction with the host's immunological response, contributes to determining the observed clinical pleiotropy and affects the efficiency of therapy applied [2]. *Leishmania* genetic diversity may also compromise vaccine development, although key antigen genes are highly conserved. Understanding leishmaniasis and the development of measures to counter its spread depend on the ability to identify *Leishmania* species and characterize genetic variants.

Multilocus enzyme electrophoresis (MLEE) is still considered by many to be the gold standard for *Leishmania* identification, but several DNA based methods have proven useful to study *Leishmania* genetic diversity [3], [4]. DNA sequencing and PCR-RFLP of *hsp70* genes have been shown to be promising for the identification of *Leishmania* parasites [5], [6], although too conserved for intra-specific diversity studies. Highly polymorphic markers, such as microsatellites, perform poorly at taxonomic levels higher than species, whilst most other genotyping methods rely on multicopy genes that are more difficult to analyze.

A standardized, sensitive, practical and reproducible typing method, such as multilocus sequence typing (MLST), must form the basis for a robust classification of *Leishmania* species, which is achievable in most laboratories even in the advent of fast and cheaper genome sequencing.

MLST, as proposed in 1998 for bacterial pathogens [7], provides a portable, reproducible, and quantitative typing system. It has since been applied to diploid organisms [8]–[10], including *Leishmania*, with ten markers described for the *L. donovani* complex [11]–[13] and four targets proposed for *L. (Viannia)* spp. [14]. MLST databases for *Leishmania* have not yet been implemented and the published number of strains and species typed is relatively low. Alongside MLST, multilocus sequence analysis (MLSA) has been shown to be a good tool for strain characterization and epidemiological surveillance, as well as population structure and evolutionary studies [9], [10], [15], [16]. The implementation of an MLST system demands careful evaluation of markers beforehand to study their diversity and phylogenetic consistency [17], [18].

We report here the evaluation of four candidate coding regions, located in different chromosomes, to be included in an MLSA system of the subgenus *L. (Viannia)*. These regions are part of the genes for the metabolic enzymes glucose-6-phosphate dehydrogenase (G6PDH, EC 1.1.1.49), phosphogluconate dehydrogenase (6PGD, EC 1.1.1.44), mannose phosphate isomerase (MPI, EC 5.3.1.8) and isocitrate dehydrogenase (ICD, EC 1.1.1.42). Three of these genes had been studied previously, but mainly for Peruvian isolates of the *L. braziliensis* complex. Herein a large number of strains and most species of the subgenus *L. (Viannia)* were included. The sequences were used to conduct haplotype analysis, including the construction of haplotype networks. Diploid multilocus analyses were performed and a concatenated tree was defined, as well as clonal complexes (CC). Using an MLSA approach, we report the genetic diversity of the four gene fragments evaluated in the *L. (Viannia)* group and demonstrate their value for taxonomic, phylogenetic and population genetic studies of *Leishmania* parasites.

## Methods

### Ethics statement

Research in this study was subject to ethical review by the European Commission and approved as part of contract negotiation for Project LeishEpiNetSA (contract 01547): the work conformed to all relevant European regulations. The research was also reviewed and approved by the ethics committee of the London School of Hygiene and Tropical Medicine (approval 5092). The *Leishmania* strains analyzed were principally strains derived from international cryobanks such as Coleção de Leishmania do Instituto Oswaldo Cruz (CLIOC) and the cryobank of the London School of Hygiene and Tropical Medicine (LSHTM). In all cases *Leishmania* were isolated from patients as part of normal diagnosis and treatment with no unnecessary invasive procedures and with written and/or verbal consent recorded at the time of clinical examination. Data on isolates were coded and anonymised. The *Leishmania* strains are deposited either at the LSHTM (n = 9) or at CLIOC (n = 87) as open data. CLIOC is a Depository Authority by the Ministry of the Environment [Fiel Depositária pelo Ministério do Meio Ambiente, MMA] (D.O.U. 05.04.2005). All samples were used for research purposes only and the data were analyzed anonymously in the scope of resolution 21 (August 31, 2006 – CGEN/MMA), for which authorization is not required.

### Samples and GenBank available sequences

Ninety six strains were obtained from frozen stocks from CLIOC (n = 87), and from the London School of Hygiene and Tropical Medicine (LSHTM) (n = 9) (Table S1). Strains were chosen to be representative of the zymodeme and geographical diversity of species of the subgenus *Leishmania (Viannia)* in South America, and in particular Brazil: *L. (V) braziliensis*, *L. (V) guyanensis*, *L. (V) naiffi*, *L. (V) lainsoni*, *L. (V) shawi*, *L. (V) utingensis* and *L. (V) lindenbergi*. Most strains had been previously characterized by MLEE [19]. DNA sequences were retrieved from GenBank of *L. (Viannia)* species: 14 of G6PD; 24 of 6PGD; 20 of MPI and 1 of ICD (Table S2). Only those presenting full coverage with the alignment of sequences obtained in the present study were included in the analysis: for G6PD: 3 *L. braziliensis*, 2 *L. guyanensis*, 1 *L. peruviana*, 1 *L. panamensis*, 1 *L. lainsoni*; for 6PGD: 10 *L. peruviana*, 07 *L. braziliensis*, 1 *L. panamensis*; for MPI: 11 *L. peruviana*, 9 *L. braziliensis*; for ICD: 1 *L. braziliensis*.

### DNA extraction and PCR

Promastigotes were grown at 25°C in Schneider's medium supplemented with 20% (v/v) heat-inactivated fetal bovine serum to a density of 1×10^9^ cells/mL (late log phase), as estimated by counting in a Neubauer chamber. DNA extraction was performed using the Wizard DNA purification Kit (Promega, Madison, USA) following the manufacturer's instructions.

The chosen loci are distributed on different chromosomes according to *L. (Viannia) braziliensis* genome sequencing data: 6PGD, on chromosome 34; G6PD, on chromosome 20; MPI, on chromosome 32; ICD, on chromosome 33.

Primers (Table 1) were designed in conserved regions of gene sequences from the published *L. major* and the *L. braziliensis* genomes in Genbank (www.ncbi.nlm.nih.gov/Genbank) to amplify sections of the coding regions of the genes that would be amenable to full sequencing using the PCR primers and that include putative species-specific polymorphisms as well as singleton SNPs. Amplification reactions had, for 50 µl total volume, 0,1 mM of each primer, reaction buffer (100 mM Tris–HCl, pH 8.8; 500 mM KCl, 1% Triton X-100; 15 mM MgCl_2_), 0.25 mM deoxyribonucleotide triphosphate (dNTPs), 0.025 U FideliTaq/GoTaq polymerase and approximately 50 ng DNA. Amplification conditions were: 94°C for 2 minutes, followed by 35 cycles at 94°C for 30 seconds, 55°C (for ICD, 6PGD and G6PD) or 58°C (for MPI) for 30 seconds and 68°C for 1 minute, with a final extension at 68°C for 5 min.

**Table 1:** Detail of target regions of each locus studied.

Locus	Gene ID*	Gene length	Amplicon size (bp)	Primer positions	Primers sequence 5′-3′
G6PD	LbrM.20.0160	1686	881	173	ATGGAAGCGTGTGATCGAAT
				1015	GGCTCAACACACTTCAGCAA
6PGD	LbrM.34.3250	1440	836	143	CTCAAGGAACATGAGCACGA
				940	TTGTCCTTGACTTGCTCACG
MPI	LbrM.32.1750	1287	681	128	GGCAAGATGTATGCGGAGTT
				770	CTCCCCAGGAACCATCTGTA
ICD	LbrM.33.2820	1278	1022	99	GAATCGGGAAGGAGATCACA
				1082	CATCATAGCCCCAGAGAGGA

Bp- base pairs; Fw - Foward; Rv – Reverse.

* related to *L. braziliensis* genome.

### DNA sequencing

PCR products were purified with the Wizard SV Clean-up System (Promega). The final DNA concentration was estimated by comparison with a DNA Ladder Marker (Promega) in 2% agarose gel. Sequencing was performed with the same primers used for amplification, using the ABI PRISM BigDyeTerminator v3.1 Cycle Sequencing Kit, and products analyzed in an automated DNA sequencer (ABI-3730). Consensus sequences were generated and edited in Phred/Phrap/Consed Version: 0.020425.c [20] from two forward and two reverse strands. Sequences with Phred values below ten over their extent were discarded and only sequence segments with values above twenty were used for contig construction. Ambiguous (heterozygous) sites were coded using the standard IUPAC codes for combinations of two or more bases. Contigs from all samples were manually assembled and aligned in MEGA4 [21]. The homologous sequences available in GeneBank for other *L. (Viannia)* isolates were obtained using the Basic Local Alignment Search Tool (BLAST) algorithm hosted by NCBI, National Institute of Health, USA (http://www.ncbi.nlm.nih.gov) and included in the alignments.

### Haplotype analyses

Haplotype reconstruction was done through DNAsp5 [22] using the PHASE algorithm, which automatically assigns a haplotype number (H) for each unique haploid sequence. Haplotype data was then used in DNAsp to calculate the synonymous and non-synonymous substitution rates and haplotype diversity (HD). Haplotype diversity (HD) was calculated as: HD = N(1−Σxi^2^)/(N−1) where xi is the haplotype frequency of each haplotype in the sample and N is the sample size. The average haplotype diversity for species was compared through ANOVA.

The discriminatory capacity of each marker employed was calculated through Simpson's diversity index (D), as follows: D = Σ n(n−1)/N(N−1), where n = the total number of strains of a particular haplotype and N = total number of strains analyzed. Phylogenetic congruence between markers was assessed by comparison of maximum parsimony (MP) and median-joining (MJ) networks generated by the Network free software [23], using individual haplotype data.

### Diploid multilocus analysis

For each sample a sequence type number (ST) was defined with each marker, which in the homozygous strains was identical to the haplotype number (H), and for the heterozygous strains was a combination of the two possible alleles. A diploid sequence type (DST) was defined for the final combination of STs of the four markers. The sequences from the four genes were concatenated using BioEdit *v7.0.9* and unique concatenated sequences were identified with the DST previously assigned. To evaluate the phylogenetic information provided by the four markers and to investigate the presence of recombination signatures, a NeighborNet network was built in SplitsTree 4.0 [24] based on genetic distances calculated according to the Kimura-2 parameter model of nucleotide substitutions from concatenated data.

ST data combinations (with the exception of heterozygous strains) were analyzed in e-BURST v.3 (http://eburst.mlst.net/v3/enter_data/single/) to define clonal complexes (CC), which are sets of related strains containing pairs of strains that share at least (L-1) identical alleles at the L loci with at least one other member of the CC.

## Results and Discussion

We report here the most comprehensive multilocus sequence analysis study to date of the subgenus *Leishmania (Viannia)*, based on fragments of four different metabolic enzyme coding genes from a wide range of species, zymodemes and geographical origins.

With the aim of developing a working MLST scheme, we selected polymorphic coding regions that could be sequenced with forward and reverse primers. The full coding regions of three of the studied genes (6PGD, G6PD and MPI) had already been sequenced and characterized for some *L. (Viannia)* strains, mainly of Peruvian *L. braziliensis* and *L. peruviana*. However, we also partially analyzed the coding region for ICD and studied a large number of strains (96), to include most known species or complexes of *L. (Viannia)*.

### Genetic diversity of the four housekeeping genes used in the MLSA of *Leishmania (Viannia)*: Polymorphic, ambiguous and multi-allelic sites

The sequences obtained for each gene varied between 589 (MPI) and 914 bp (ICD) (Table 2) and covered between 38% (G6PD) and 71% (ICD) of the corresponding gene. Sequences were deposited in GenBank (www.ncbi.nlm.nih.gov/Genbank) with accession numbers JN996517–JN996708 and JQ181608–JQ181801 and are also available at the CLIOC website (htpp://clioc.fiocruz.br).

**Table 2:** Characteristics of each sequenced region for the 96 strains used in the present study.

Gene	Length	PS	PI (bp)	Singletons	Ambiguous sites	
	(bp)	(bp)	(% of length)	(% of length)	Number of sites (% of length)	Number of isolates (% total strains)
G6PD	683	50	46 (6.7%)	4 (0.6%)	6 (0.9%)	8 (8.3%)
6PGD	716	63	60 (8.4%)	1 (0.1%)	5 (0.7%)	9 (9.4%)
MPI	589	50	49 (8.3%)	1 (0.2%)	2 (0.3%)	2 (2.1%)
ICD	914	78	76 (8.3%)	2 (0.2%)	6 (0.7%)	10 (10.4%)

Percentages are relative to the total number of sites (% of length) or strains (% of total strains). PS – Polymorphic sites; PI - parsimony informative sites.

Gene fragments had 50–78 polymorphic sites (respectively, 7.3–8.7%) for the strains studied here, of which 46 (G6PD) to 76 (ICD) were parsimony informative (PI) sites (Table 2). 6PGD had the highest percentage of PI sites (8.4%), with the lowest found in G6PD (6.7%). The studied G6PD region had a much higher number and percentage of singletons, 4 (0.6%), in relation to the respective total template length than the other three gene regions (0.1–0.2%; Table 2).

**Figure 1:**
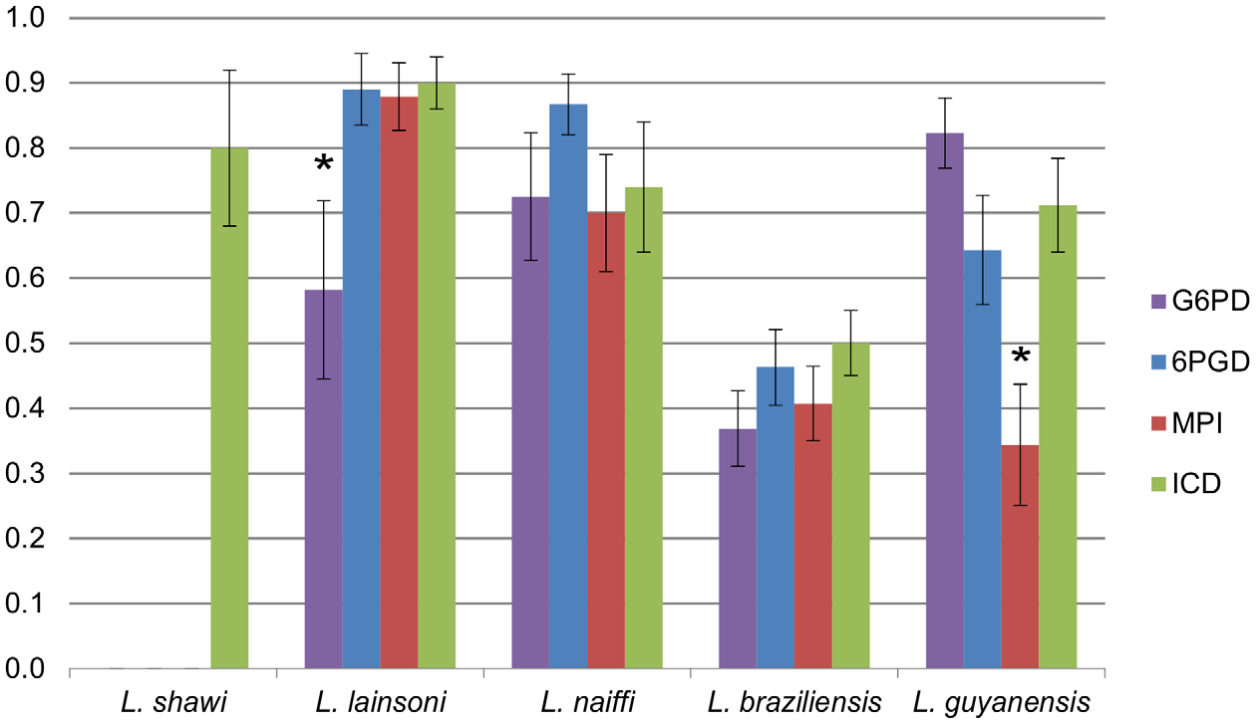
Comparison of haplotype diversity values of each locus within species groups. ICD was the only polymorphic marker for *L. shawi* strains. * G6PD and MPI were the least polymorphic markers (P<0.001) within species for *L. lainsoni* and *L. guyanensis* respectively.

The four sequenced fragments, representing different loci, had different degrees of diversity between species groups as seen by the haplotype diversity (Figure 1) and are thus suitable to be used in combination for intra- and inter-specific inferences. Although theoretically including more loci improves discriminatory capacity, it has been described that increasing the number of genes above four did not increase discrimination of MLST [18]. Furthermore, the index of diversity [25] for the targets included in the present study implies that the typing results can be interpreted with confidence.

Overall, after including published gene sequences for the same regions as studied here (except for ICD, which had no available sequences) gene fragments provided 51 (MPI) and 64 (6PGD) PI sites (Table 3).

**Table 3:** Characteristics of each sequenced region for all available *L. (Viannia)* (retrieved from GenBank plus the 96 strains included) and for *L. braziliensis* complex samples.

Gene		Length (bp)	PI*	Singletons	Ambiguous sites	
			(% of length)	(% of lentgh)	Number of sites	Number of isolates
G6PD	*L. (Viannia)*	683	54 (7.9%)	4 (0.6%)	6	8
	*L. braziliensis*		11 (1.6%)	0	2	5
6PGD	*L. (Viannia)*	716	64 (8.9%)	1 (0.1%)	5	9
	*L. braziliensis*		27 (3.8%)	0	3	5
MPI	*L. (Viannia)*	589	51 (8.7%)	1 (0.2%)	2	2
	*L. braziliensis*		17 (2.9%)	1 (0.2%)	3	3

Percentages are relative to the total number of sites (% of length).

* PI - parsimony informative sites.

Most polymorphic sites were bi-allelic, although sites with three variants occurred in three genes, G6PD, 6PGD and in ICD (Table S3). Recently, the genome plasticity of some *Leishmania* species was analyzed [26]. The evaluation of the *L. braziliensis* genome showed that 30 of 35 chromosomes are clearly trisomic; three are tetrasomic (chromosomes 4, 5 and 29) and one hexasomic (chromosome 31). Moreover, the same study showed that multicopy genes are found preferentially on non-supranumerary chromosomes. Such peculiarities are of great importance in all molecular analyses proposed for the *Leishmania* genus. In the present work, all markers used are located on different chromosomes, which are described as non-supranumerary [26]. However, the description of a trisomic genome for *L. braziliensis* indicates that multiallelic possibilities might be quite frequent when polymorphic sites are analyzed in DNA sequences of *Leishmania (Viannia)* species. Moreover, gene copy numbers might also change between species and generations of the same strain.

Twenty-six isolates had at least one locus with double peaks in the chromatograms (Table 2 and Table S4). The number of sites showing double peaks was similar in three gene regions, but much smaller for MPI, which also had a much smaller number of strains with double peaks in their sequences compared to the other targets (Table 2). Different strains presenting double peaks at the same site were observed in all markers, except MPI (Table S4).

Although most strains were homozygous, almost a third had at least one locus with double peaks, except *L. shawi*. MPI was the most homozygous gene, even though it was not the most conserved. All the other markers presented more than one double peak per strain and more than one strain with double peaks. It is possible that this is a sampling effect of the sequenced region in MPI, or that gene conversion in this gene is higher [27].

The presence of ambiguous sites and possible parental alleles among the samples studied strongly suggests real heterozygosis and also the occurrence of some level of recombination. Ambiguous sites were observed in twenty positions over the four gene fragments analyzed (Table S4), and putative homozygous parental were observed in eight of them. Many strains had two or more heterozygous sites. Heterozygosis can be caused by mutation in one allele but it can also be caused by genetic exchange between strains with different alleles. Mutation is more likely for single heterozygous sites but recombination is a more parsimonious explanation for two or more sites [28]. The majority of heterozygous alleles among our sample presented just one ambiguous site. Exceptions were encountered in one *L. braziliensis* strain showing HP 8/17 in 6PGD and 6/11 in ICD, with two and three ambiguous sites respectively. This observation reinforces the fact that real heterozygosis might be occurring, because these two loci are on different chromosomes and in both sequence alignments at least two ambiguous sites were observed in that sample. Moreover, after determination of the minimum number of recombination events by using DNAsp, recombination between sites was detected for the four markers, with highest frequency for 6PGD and least for G6PD (data not shown).

The assumption that ambiguities in the chromatograms are the result of heterozygosis can only be reliably postulated if the target is a single copy gene. In our case, the four selected genes are single copy genes, as estimated by in silico analysis of the four available *Leishmania* species reference genomes (data not shown). Heterozygous samples would need confirmation through biological cloning of isolates, to exclude polyclonal populations. The strains we have used were not cloned, except for the reference strains. As far as we know, none of the studies aiming to construct an MLST system for *Leishmania* have used biologically and/or genetically cloned the samples. Following an overview of sample profiles, without cloning, if ambiguous sites are detected, the strains containing them should be selected for deeper analysis and cloning.

### Species-specific SNPs

Putative species-specific SNPs, as determined through the data presented here and available in GenBank, were detected for *L. guyanensis* in 6PGD (A105); for *L. naiffi* in MPI (15T, 82A, 98A, 270A, 306C, 498C, 543T) and G6PD (320C, 341C, 432A, 467A); for *L. lainsoni* in MPI (33G, 105G, 294T) and G6PD (116A, 239T, 247G, 326A, 464C, 482C, 572A, 577T) and for *L. shawi* in MPI (135G). No species-specific SNPs were detected for *L. braziliensis*. ICD was the only marker that did not present species-specific SNPs.

In a previous study, species-specific SNPs for all species, including *L. braziliensis* were shown [14]. This incongruence between the two studies might be a consequence of differences in the gene fragments analyzed as well as in the strains studied. SNP markers should thus be used for species identification with great care, even when more strains are studied in future. Either full sequences should be obtained, or a large panel of SNP markers should be used for reliable identification and characterization.

### Identification of assorted *Leishmania (Viannia)* haplotypes

**Figure 2:**
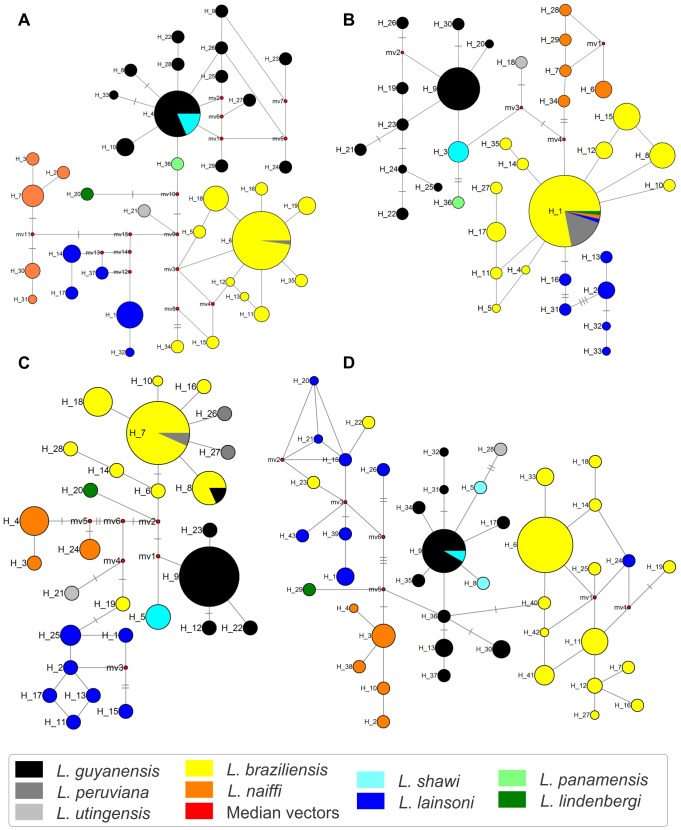
Maximum parsimony-based haplotype networks. A) G6PD; B) 6PGD; C) MPI; D) ICD. The 96 strains selected for the study (Table S1) plus sequences of *Leishmania* (*Viannia*) strains retrieved from GenBank (Table S2) were included. Haplotype frequency is represented by the size of each node and the numbers of polymorphisms are indicated in the branches by dashes: one = 02 to 05 polymorphism; two = 06 to 10; three = more than 10 polymorphisms. Each species, considering the MLEE characterization, were assigned by different colors as coded by the legend.

Here we found a large number of unique haplotypes, which are likely to be rare or recent in the population. In contrast, some haplotypes were shared across species, such as haplotype H1 of 6PGD, which was detected in strains from different species: almost all *L. braziliensis* (n = 40), nine *L. peruviana* as well as two previously published *L. braziliensis* sequences, one *L. lainsoni* (IOCL 2500 from Acre) and one *L. naiffi* (IOCL 855 from Amazonas) strain. This could be due to recombination or convergent evolution, but it could also represent the most likely ancestral haplotype, as depicted from the haplotype network constructed for this gene (Figure 2). Remarkably, those species present different electrophoretic mobility for the 6PGDH enzyme system. Such incongruence might be due to the fact that the entire coding region was not sequenced here, so sequence sections coding for differences in MLEE might not be present in the current analysis, or it might be due to post-transcriptional and post-translational modifications [28].

Upon haplotype assignment it was found that among the 96 studied strains there were 25 (MPI) to 43 (ICD) different haplotypes, of which 16 (MPI) to 31 (ICD) were represented by only one strain. Nevertheless, ICD had the highest percentage of exclusive haplotypes per strain (72.1%) and G6PD had the lowest (63.6%; Table 4).

**Table 4:** Haplotype analysis per marker for the 96 strains included in the present study.

Gene	Length (bp)	N° of different alleles	HD*	N° unique alleles
G6PD	683	33	0.78	21 (63.6%)
6PGD	716	34	0.76	22 (64.7%)
MPI	589	25	0.77	16 (64%)
ICD	914	43	0.85	31 (72.1%)

Percentages are relative to the number of different alleles.

* HD - haplotype diversity.

Strains identified as *L. braziliensis* represented more than 50% of the strains studied. The greatest number of haplotypes was observed within the *L. braziliensis* group for all markers except G6PD for which the greatest number of haplotypes was observed within the *L. guyanensis* group (Table 3). Around 30% of the haplotypes in each gene were detected in *L. braziliensis* (Table 3). However, almost no singletons and no species-specific SNPs were observed among the *L. braziliensis* strains, but some ambiguous sites were seen (Table S4). *L. utingensis* and *L. lindenbergi* had unique haplotypes for all markers, with the exception of 6PDG for *L. lindenbergi*, as mentioned above.

Regarding G6PD, 12 haplotypes were shared by at least two strains. The most common haplotype, H6, was found in 45 strains, characterized as *L. braziliensis* or *L. peruviana*. Four additional haplotypes were found in previously published sequences, one in *L. panamensis* (H36), two *L. braziliensis* (H34, from Peru, and H35, from Brazil) and one *L. lainsoni* (H37, from Peru). One published *L. guyanensis* sequence had haplotype H4, the most common *L. guyanensis*/*L. shawi* haplotype in this analysis (Table S2). Except for *L. shawi* and *L. peruviana*, which shared haplotypes with *L. guyanensis* and *L. braziliensis* respectively, the network depicted from this target presented the best clustering accordingly to species (Figure 2A).

Concerning 6PGD, haplotype H15 was only found in one isolate of *L. braziliensis* from Acre, northern Brazil, but it was identical to four previously published *L. braziliensis* strains from Peru. Similarities among strains from these two adjacent areas were reported previously based on MLMT [29], corroborating the suggestion that a geographic cluster and probably a hierarchical population structure of *L. braziliensis* exist in this area. *L. guyanensis* had 10 haplotypes, of which H9 was the most frequent, and *L. shawi* had one distinct haplotype (H3). Only two 6PGD haplotypes found in previously published sequences (one *L. braziliensis* from Peru and one *L. panamensis*) were not detected among the strains studied here, indicating a good coverage of haplotype diversity by our study.

The two most frequent alleles observed, corresponding to 6PGD H1 and H8 (Figure 2B), were also observed combined in one heterozygous *L. braziliensis* strain (IOCL 2833). These haplotypes were also observed in one (H1; IOCL 2494) and two (H8 IOCL 918 and 2538) other heterozygous strains, combined with other haplotypes (Table S4). Some 6PGD haplotypes were unique to heterozygous strains, as H4/H5 and H30/H31 (Table S1).

MPI *L. braziliensis* haplotype H8 was also found in one *L. guyanensis* strain. Haplotype H7 was the most common in *L. braziliensis* (41 strains) and was also found in eight previously published sequences of *L. peruviana* and eight of *L. braziliensis* (Table S2). MPI was previously reported as a good target to discriminate between *L. braziliensis* and *L. peruviana* [14], [30]. Our conflicting results could be related to differences in the fragment regions analyzed, or might reflect a bias in the sample analyzed previously. Two published sequences of *L. peruviana* strains presenting an ambiguous site had the most common H7 and a new haplotype 26 after allele reconstruction. One available *L. peruviana* and one *L. braziliensis* sequence from Peru presented additional haplotypes H27 and H28, respectively (Figure 2C; Table S2).

The greatest number of haplotypes was found in ICD, for almost all species, although there were similar levels for G6PD and 6PGD in *L. naiffi*. ICD H6 was by far the most common *L. braziliensis* haplotype, including one previously published sequence (Table S2). ICD H9, the most common *L. guyanensis* haplotype, was also found in one *L. shawi* (IOCL 3200) (Figure 2D; Table S1).

Overall, and including published gene sequences for the same regions as studied here, there are 28 to 43 different described alleles in *L. (Viannia)*, of which 11 to 16 are in the *L. braziliensis* complex.

### Haplotype diversity and haplotype network selection of marker combinations for resolution of inter- and intra-species relationships

Overall, haplotype diversity (HD) was higher for ICD (0.85), similar among the other gene regions (0.76–0.78) and as compared in ANOVA not significantly different (P>0.01). However, upon analysis by species, MPI was the least polymorphic marker for *L. guyanensis* (P<0.001) and G6PD was the least polymorphic locus for *L. lainsoni* (P<0.001). Among the three *L. shawi* isolates, ICD was the only polymorphic marker (Figure 1).

The Simpson index of diversity (D), which provides an indication of the discriminatory capacity of each marker, was similar for MPI, G6PD and 6PGD, (0.77, 0.77 and 0.78 respectively), but higher for ICD (D = 0.89). Analyses of the discriminatory capacity indicate a high level of strain discrimination, of almost 90%, using these four loci. However, higher values were observed when applied to *L. braziliensis* and *L. guyanensis* (96% and 100%, respectively). Other species were represented by few strains and were not analyzed. This suggests that, even though the number of markers is smaller than that usually used in MLST (7), these four markers are sufficient for studies in *L. (Viannia)*. However, more detailed population genetics studies may require more markers. Regarding the discriminatory power of each marker, the Simpson index showed that ICD had the highest diversity. Indeed, this is a very polymorphic locus in MLEE, which is able to detect intra-species variation [19].

MPI was here found to be a good marker to distinguish between the species, although HP8 was shared among *L. braziliensis* and one *L. guyanensis* strain. This enzyme is used as a marker to differentiate *L. peruviana* from *L. braziliensis*, which was supported by DNA sequencing in a previous study [14] by the detection of a specific SNP, as well as others useful to differentiate closely related *L. (Viannia)* species. We could not confirm this, given that our sequences were shorter and did not include that SNP locus.

Maximum parsimony-based haplotype networks built for each gene (Figure 2) showed that species, as identified by MLEE, clearly formed separated clusters in each gene network, with a few exceptions confined to some strains that for some markers were not grouped in their species haplogroups. Reticulate patterns were observed in some clusters for all loci studied. Even with markers for which *L. shawi* presented different haplotypes from *L. guyanensis*, these two species always clustered close together.

Haplogroups were, in general, consistent with the species (color-coded nodes), although exceptions occurred for all markers: G6PD (H4 and H6; Figure 2A); 6PGD (H1; Figure 2B); MPI (H7, H8 and H19; Figure 2C) and in the ICD network (H9, H22, H23, H24; Figure 2D). A single haplotype MPI (H19) comprising an *L. braziliensis* strain (IOCL 2541, *L. braziliensis* from Pernambuco) was at the base of the *L. lainsoni* cluster, but for all the other markers this strain clustered within the *L. braziliensis* species group. One *L. lainsoni* strain (IOCL 2500 from Acre) was part of the *L. braziliensis* cluster for ICD (H24) and 6PGD (H1). Conversely, the *L. lainsoni* cluster included two haplotypes exclusively from *L. braziliensis* strains (haplotypes H22 and H23, IOCL 2498 and IOCL 2499 respectively, from Acre), even though both clusters were located in opposite sides of the network.

The most frequent haplotypes (node size) were often the founding haplotype for a given haplogroup, as clearly observed for *L. braziliensis* (MPI H7, ICD H6, G6PD H6, 6PGD H1) and *L. guyanensis* (MPI H9, ICD H9, G6PD H4, 6PGD H9). Eighteen out of 55 *L. braziliensis* strains were frequently observed in the most common haplotypes, but for *L. guyanensis* the composition of the most common haplotypes was different between the markers. The most frequent haplotypes were also usually those in which inter-specific sharing of sequences was observed (Figure 2).

*L. guyanensis* formed a diverse cluster, whereas *L. shawi* strains presented a profile coherent with a subpopulation of the *L. guyanensis* group for all markers, commonly sharing the most common *L. guyanensis* haplotype or differing from it in at most two polymorphisms (Figure 2C).

Although we used a relevant number of strains, the data analysis by Network software generated median vectors. The presence of median vectors in the networks might indicate that: i) intermediate haplotypes were present in lost populations; ii) haplotypes from populations that not included in this analysis; iii) the ancestors of these strains suffered rapid adaptive evolution with expansion of these extant strains. Therefore, even without sequencing strains representing all genetic diversity, statistical tools may predict the variability.

Neighbour Joining (NJ) trees were built for each marker to evaluate the phylogenetic relationship between the haplotypes. Almost no incongruence was observed between the markers. Four monophyletic groups were clearly observed for each marker, representing basically *L. lainsoni* and *L. naiffi*, the most divergent groups, and *L. braziliensis* and *L. guyanensis* (in this case, including *L. shawi*), which were very closely related for any marker (data not shown), corroborating the genetic distance MLEE-based tree [19]. *L. lindenbergi* (except in 6PGD) and *L. utingensis* were each in a separate and independent branch, but grouping closer to *L. naiffi* and *L. guyanensis* respectively.

Available sequences of *L. peruviana* for G6PD, 6PGD and MPI, and for *L. panamensis* for G6PD and MPI, were included in the respective network constructions. *L. peruviana* sequences either presented the most frequent haplotype for *L. braziliesnis* strains (in G6PD H6, 6PGD H1 MPI H7; Figure 2) or differed from it in one polymorphic site (in the MPI H26 and H27; Figure 2C; Table S2). A previous study using random amplification of polymorphic DNA (RAPD) and MLEE [31] reported that *L. peruviana* and *L. braziliensis* corresponded to two closely related, but distinct monophyletic lines, which was not corroborated by *hsp70* gene sequence analysis [32]. *L. panamensis* sequences presented new haplotypes (G6PD H36 and 6PGD H36) within the *L. guyanensis* and *L. shawi* haplogroup. Previously [33] MLEE and RAPD analysis questioned the distinction between *L. panamensis* and *L. guyanensis*, since data did not indicate distinct monophyletic lines. In individual NJ trees for the gene fragments studied here (data not shown), the *L. panamensis* sequences clustered within the *L. guyanensis*/*L. shawi* group and *L. peruviana* clustered within the *L. braziliensis* group. It was not possible to include *L. panamensis* and *L. peruviana* in the final MLSA conclusions since there were no available sequences for all genes studied. Furthermore, more strains from both species should be sequenced for the four gene targets to infer properly on the monophyletic origin of them.

### Concatenated NeighborNet confirms species-specific clusters and suggests relatively frequently recombination occurring in *L. braziliensis* and *L. guyanensis*

Among the 96 *L. (Viannia)* strains, 75 final diploid sequence types (DSTs) were assigned. Although we detected a high number of DSTs, many DSTs are unique, while others are more prevalent, widely distributed and presenting temporal stability, which might reflect limited genetic recombination involving these DSTs [17]. The only species with strains sharing the same DST was *L. braziliensis*. DST12, for example, was found in 18 strains of *L. braziliensis* from different Brazilian endemic regions related to the Atlantic rain forest, except one, and present zymodeme diversity although most are classified in zymodeme 27 (Table S1). DST12 is not only the most frequent but also shows temporal stability, as the strains included in this DST had been isolated between 1987 and 2001. Strains typed as DST12 were isolated from patients presenting distinct clinical manifestations. This raises the intriguing proposition that the apparent dominance of DST12 in endemic locations associated with urban areas of the Atlantic rain forest region may be a consequence of higher fitness of this DST to the modified environment.

Three other *L. braziliensis* DSTs (16, 27, and 35) comprised two or three strains. All the other *L. braziliensis* were assigned to unique DSTs (n = 30). DST16 comprised two heterozygous *L. braziliensis* strains from Peru, whilst DST27 comprised two strains (zymodemes 27 and 74) from different localities (Pernambuco and Bahia, respectively), and DST35 three strains from the same zymodeme (IOC/Z27) and geographic origin (Bahia, Northern Brazil).

This demonstrates that the MLSA approach allows both detection of different genotypes and the level of separation between strains through the number of polymorphic sites.

**Figure 3:**
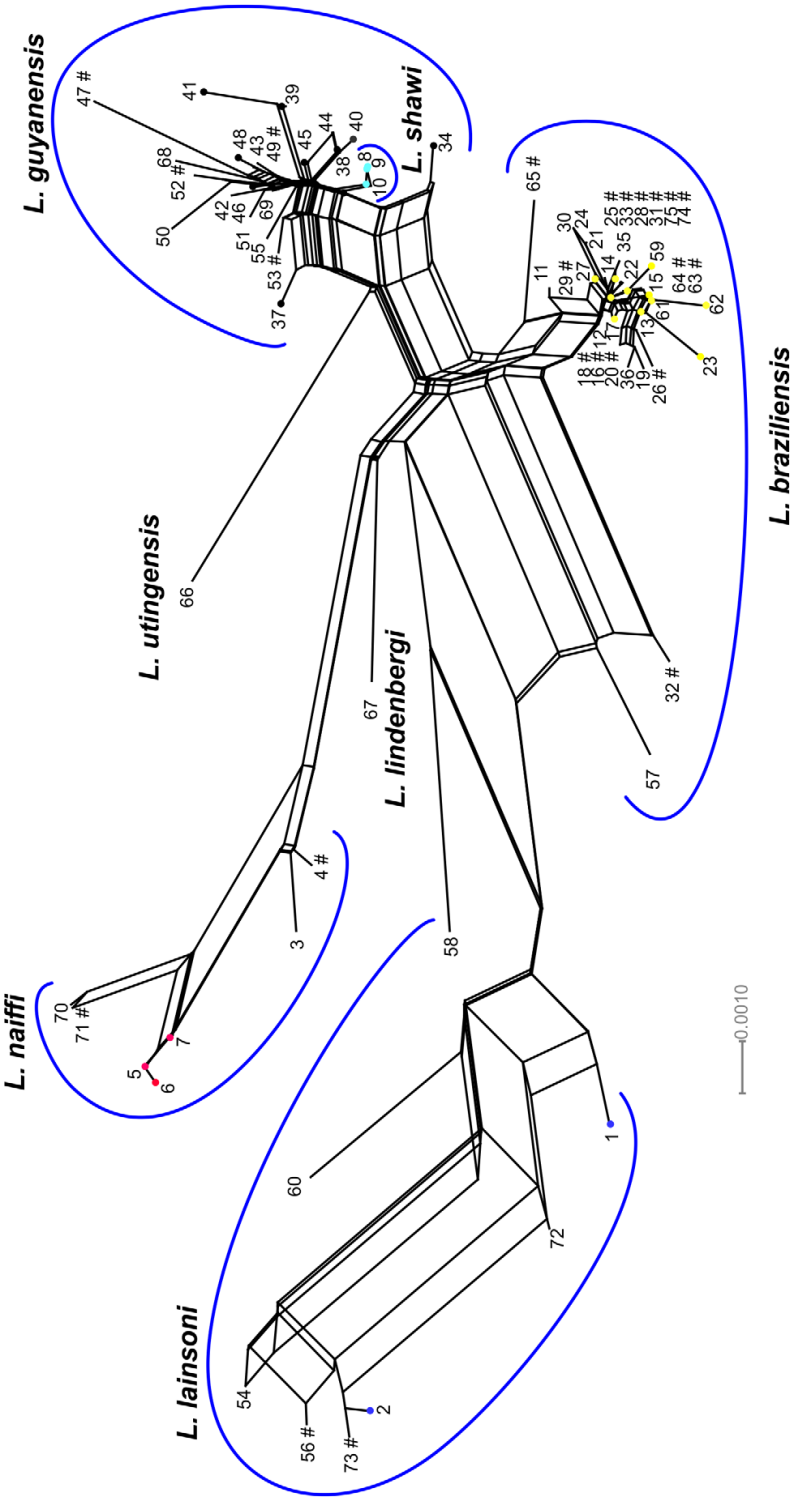
NeighborNet graph based on concatenated sequences of four gene fragments of *Leishmania (Viannia)* subgenus. A bushy network structure is observed indicating homologous recombination. # = DSTs presenting ambiguous sites (possible heterozygous) in at least one locus; DSTs gathered in clonal complex are represented by different colored circles depending on the species: yellow - *L. braziliensis*; black – *L. guyanensis*; light blue – *L. shawi*; blue – *L. lainsoni*; red – *L. naiffi*. Singletons are represented by DST number only.

A NeighborNet network was obtained with the concatenated sequences represented by the DSTs (Figure 3). The clusters were in agreement with MLEE for species groups, with the exception of *L. shawi*, which clusters together with all *L. guyanensis* strains. The reticulate aspects of the *L. guyanensis* group suggest recombination events occurring among the strains, including *L. shawi* (Figure 3). This same aspect was observed for the *L. braziliensis* cluster, but two strains were more divergent (IOC/L2498 and IOC/L2499, DSTs 32 and 57). Both were isolated from Acre state, a region bordering Peru. Recently we have demonstrated that these two strains clustered together with *L. peruviana* and Peruvian *L. braziliensis* by microsatellite typing [29]. However, upon removal of ICD sequences from this analysis these two strains grouped very closed to the other *L. braziliensis* strains (data not shown).

It is clear that *L. braziliensis*, *L. guyanensis*, *L. naiffi* and *L. lainsoni* all represent distinct species, forming monophyletic groups in the NeighborNet. *L. lindenbergi* and *L. utingensis* were placed close to the monophyletic groups corresponding to *L. naiffi* and *L. guyanensis* respectively, corroborating MLEE and MLMT data (unpublished). More isolates from both species should be studied to infer their taxonomic status. However, it is important to mention that these two species shared no alleles with all the other species/strains, except for *L. lindenbergi*, which shared alleles in 6PGD with *L. braziliensis* (Figure 2B). The estimate of the average genetic distance between *L. lindenbergi* or *L. utingensis* and the other *L. (Viannia)* species is comparable to those observed between each of the species analyzed. The highest values were observed between *L. lainsoni* and any other species.

Corroborating the results observed for the haplotype network, *L. naiffi* and *L. lainsoni* were the most divergent and the large splits observed suggesting no influence of genetic exchange. DST58, represented by a single *L. lainsoni* strain was positioned in a split between *L. lainsoni* and *L. naiffi*. The extensive reticulation suggests that recombination has occurred relatively frequently in *L. braziliensis* and *L. guyanensis*, as proposed by other authors [34]. As expected, *L. braziliensis* and *L. naiffi* presented higher within group average genetic distance values than *L. guyanensis*, even including *L. shawi* strains in the *L. guyanensis* group, corroborating previous studies on the genetic diversity of both *L. braziliensis* and *L. naiffi* [35], [36], [37], [38]. However, as far as we know, this is the first report of a high level of genetic diversity within *L. lainsoni*. This was the species giving the highest within group average genetic distance value, although it consists of only two zymodemes. Reports on the occurrence of *L. lainsoni* and *L. naiffi* in distinct endemic areas have constantly increased, indicating that both species are adapted to several environments where different sand fly species participate in parasite transmission. Notably, *L. lainsoni* was the most divergent species, followed by *L. naiffi*, which might contribute to the widespread dispersion of these two species [39]–[41].

### BURST analysis contributes to the understanding of relatedness among DSTs

Considering that a large number of unique DSTs were detected, the BURST algorithm was employed to evaluate the relationships between 52 homozygous DSTs, which corresponded to 70 strains. Here we applied eBURST to analyze the allele profiles as an efficient method to recognize natural discontinuities that would merit taxonomic status as species or subspecies. The BURST algorithm first identifies mutually exclusive groups of related genotypes in the population (typically an MLST database), and attempts to identify the founding genotype (sequence type, ST) of each group. The algorithm then predicts the descent from the predicted founding genotype to the other genotypes in the group, displaying the output as a radial diagram, centered on the predicted founding genotype. The procedure was developed for use with the data produced by MLST (STs and their allelic profiles).

**Figure 4:**
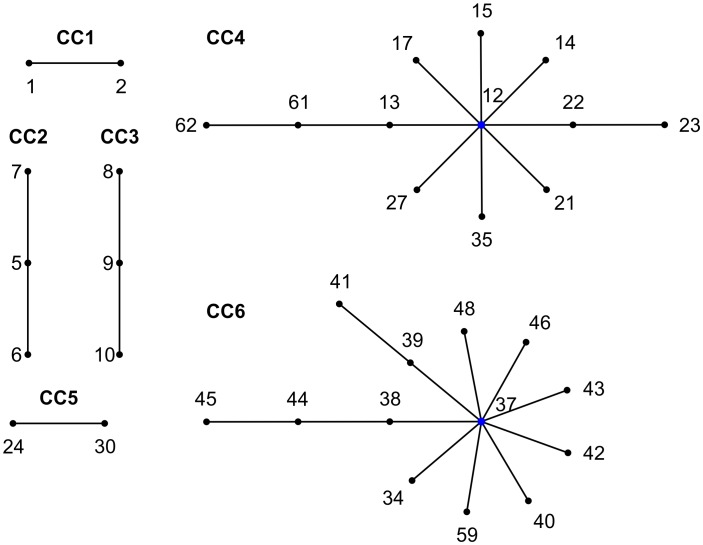
eBURST diagrams of analyzed *Leishmania (Viannia)* strains. Six clonal complexes were found: CC1: *L. lainsoni*; CC2 *L. naiffi*; CC3 *L. shawi*; CC4 *L. braziliensis*; CC5 *L. braziliensis*; CC6 *L. guyanensis*. Predicted founders were identified for CC4 (DST12) and CC6 (DST37; IOC/L565). Only homozygous DSTs were used (Table S1).

The clonal complex model has proved to be valuable in analyzing many MLST datasets, making the results more amenable for epidemiological analysis. A clonal complex (CC) comprises genetically related, but not identical, organisms. In this study six CCs were observed (Figure 4); each one represented by strains classified as the same *Leishmania* species. Strains typed as *L. braziliensis* were grouped in two clonal complexes. The CCs observed were: CC1, *L. lainsoni* (comprising 2 of 6 strains; 2/6); CC2, *L. naiffi* (3/5); CC3, *L. shawi* (3/3); CC4, *L. braziliensis* (30/37); CC5, *L. braziliensis* (2/37); CC6, *L. guyanensis* (13/17). CC6 included 13 *L. guyanensis* DSTs from 17 analyzed, indicating DST37 as the predict founder genotype. CC4 included 12 out of 19 *L. braziliensis* DSTs analyzed and the predicted founder genotype was DST12, the most common DST observed for this species.

Most of the isolates were not grouped in any clonal complex, showing a high prevalence of singletons (n = 15; 4 *L. lainsoni*, 2 *L. naiffi*, 5 *L. braziliensis*, 4 *L. guyanensis*), differing at two or more loci from all other DSTs (Table S1), typical of populations with a high rate of recombination relative to mutation and for which eBURST does not reliably indicate ancestry [42]. Although eBURST does not contribute to phylogenetic analysis in *Leishmania*, this is a helpful clustering tool to analyze MLST results from this organism, which minimizes the differences observed between each *Leishmania* strain. For example, although all *L. guyanensis* strains were assigned to a distinct DST, almost all of them clustered in the same clonal group (CC5), corroborating the already demonstrated homogeneity of the *L. guyanensis* population in Brazil [19] and contrasting with the results with microsatellites showing diversity within the *L. guyanensis* complex [34]. We cannot infer the dispersion capacity of this group, but temporal stability is certainly a characteristic of this clonal complex.

Another scenario was observed for *L. braziliensis*. Out of 55 *L. braziliensis,* we obtained groups of 18, 3, 2 and 2 strains that were assigned to the same DSTs. However, two clonal complexes were observed for this species, one represented by 57.5% of the strains analyzed (CC4), the other one by only two strains (CC5) and 27.5% representing singletons. This result would fit a hypothesis of *L. braziliensis* having better fitness than *L. guyanensis*, leading to an improved dispersion. However, more *L. guyanensis* isolates from other regions not located in Brazil and its ‘sister’ species, *L. panamensis*, would benefit this discussion.

Although the main purpose of this study was not to investigate the epidemiological findings from MLSA of *L. (Viannia)*, it is important to mention some aspects. The capacity of dispersion and adaptation of *L. braziliensis* clonal complex CC4 is evident as well as its temporal stability. Although formed by only two *L. braziliensis* strains, CC5 represents an interesting clonal complex. The two strains were isolated from the same locality, a municipality in Pernambuco state, in northeastern Brazil, where a unique zymodeme is found. Furthermore, it is interesting that a high level of genetic diversity was already demonstrated for the *L. braziliensis* population in Pernambuco, depicted by MLEE analysis. It seems that in this region two transmission cycles co-exist; involving or not involving the sylvatic environment [35], [43]. This could influence or favor the maintenance of two clonal complexes in the area.

The putative founder for the clonal cluster CC4 (*L. braziliensis*) and CC6 (*L. guyanensis*) were determined. However, it is not possible to infer if they represent the ancestor of each group. In fact, for *L. braziliensis* CC4 the putative founder is the most prevalent DST, but for *L. guyanensis* this is not true as for this species each strain was assigned as one DST.

**Figure 5:**
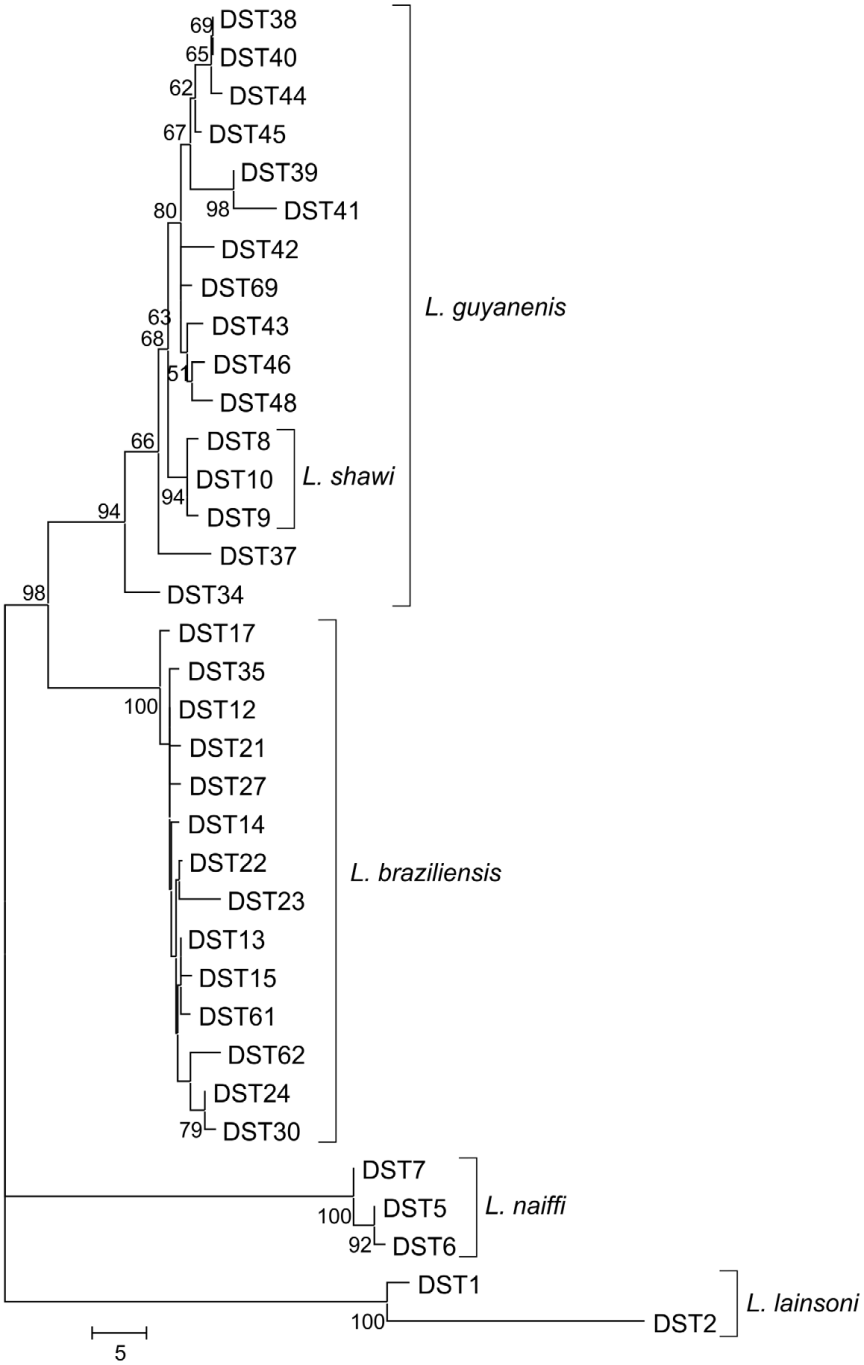
Phylogenetic relationship of *Leishmania (Viannia)* inferred from concatenated sequences of G6PD, 6PGD, MPI, ICD fragments. The phylogenetic tree was constructed using number of differences and the NJ method. Only DSTs comprised in any clonal complexes (Figure 4 and Table S1) were included. Bootstrap values (percentages of 500 replicates) above 50% are indicated at the nodes.

To evaluate the robustness of each CC and the relationship between them, a NJ tree was built using the concatenated sequences of DSTs included by eBURST in clonal complexes, excluding the singletons (Figure 5). Clusters with high bootstrap supports were observed, corresponding to each CC. DST24 and DST30, from CC5, were the only two DSTs inside the *L. braziliensis* cluster forming a group supported by the bootstrap value. Inside the *L. guyanensis* group a high supported cluster was observed composed of all CC3 DSTs, corresponding to *L. shawi*. Low bootstrap values were observed among almost all DSTs from CC4 and CC6, corroborating the fact that they belong to the same complex. Evidence was again found for the divergence of *L. lainsoni* and *L. naiffi* clonal complexes.

### Concluding remarks

The results obtained here strongly supported the established taxonomy of *L. (Viannia)*, considering the species that have been found in circulation in Brazil. Specifically, our data support monophyly of all but one Brazilian *L. (Viannia)* species analyzed here and highlight the close relationships between *L. braziliensis* and *L. guyanensis* and the recombination events occurring in both species. Some aspects merit special mention, however. The taxonomic validity of *L. shawi* has been questioned and indeed the markers studied here suggested that *L. shawi* isolates were closely related to or were part of the *L. guyanensis* group. The same was observed in the NeighborNet network with concatenated sequences. However, the eBURST analysis indicated that these isolates form a distinct clonal complex from *L. guyanensis*, although closely related as observed in the NeighborNet network. Recently, *hsp70* gene sequence analysis has indicated that *L. shawi* is a subgroup of *L. guyanensis* [5]. In addition, *L. shawi* and *L. guyanensis* had the lowest average genetic distance of the *L. (Viannia)* studied here. Our results, thus, indicate that *L. guyanensis* has different clonal populations, such as those observed for *L. braziliensis*, with *L. shawi* being one of them.

It is noteworthy that the MLSA clades derived here are in good agreement with MLEE clusters reported previously. Taken together, our data point to the combined four gene scheme used here as a reasonable approach that provides extensive differentiation and offers evolutionarily accurate clustering. The analysis performed herein should be extended to species other than those studied here and should be used as a starting point to develop an MLST scheme for *Leishmania* spp. genetic typing. While providing complete genome sequences is not possible as a routine approach, MLST generates evidence for similarities and differences between *Leishmania* species and/or strains, offering a number of advantages over most typing methods and providing results helpful for taxonomic, population genetics, evolutionary and, in consequence, epidemiological studies [4].

Such data are likely to revolutionize the systematics of *Leishmania*, consolidate our view of what constitutes a *Leishmania* ‘species’, provide evidence concerning the epidemiology of these pathogens and might even be applicable directly to clinical samples.

Definition of *Leishmania* species and knowledge of the genetic structure of all *Leishmania* species will provide a useful framework for exploring the evolutionary dynamics and phylogenetic distribution of relevant strain properties. Recognizing the urgent need for a standardized globally acceptable species definition and typing method for *Leishmania*, we are now sequencing other genes and including more species and strains in our analysis aiming to propose that species within the *Leishmania* genus could be defined as a group of strains that share a determined level of similarity in the concatenated nucleotide sequences of the genes selected. To achieve this, establishment of a consensus MLST gene set that provides optimum differentiation for *Leishmania* species and/or strains is required.

## Supporting Information

Table S1 **Taxonomic and collection data (clinical, geographical, biochemical and molecular Information) for** ***L. (Viannia)*** **strains used for MLSA.**
(DOCX)

Table S2 **Sequences retrieved from GenBank and those included in the analysis performed in the present study, indicating the assigned sequence type (ST) for each marker.**
(DOCX)

Table S3 **Multi-alleles sites observed for each fragment-gene alignment and the distribution of each allele in the studied strains.**
(DOCX)

Table S4 **Strains presenting ambiguous sites (IUPAC symbols) for the targets with the respective site position in length alignment and the most common nucleotide.**
(DOCX)
