# Supplementary material for: New Insights on Taxonomy, Phylogeny and Population Genetics of Leishmania (Viannia) Parasites Based on Multilocus Sequence Analysis
Source: PLoS Negl Trop Dis. 2012 Nov 1;6(11):e1888. doi: 10.1371/journal.pntd.0001888 (PMC3486886; doi:10.1371/journal.pntd.0001888)
Supplement: Table S1 — Taxonomic and collection data (clinical, geographical, biochemical and molecular Information) for L. (Viannia) strains used for MLSA. (DOCX) [file pntd.0001888.s001.docx]

**Table S1: Taxonomic and collection data (clinical, geographical, biochemical and molecular Information) for *L. (Viannia)* strains used for MLSA.**

| **IOC/L** | **Species** | **International code** | **Geographic origin (Brazilian state)** | **Clinical form** | **Zymodeme** | **MPI** | **G6PD** | **6PGD** | **ICD** | **DST** | **CC** |
| --- | --- | --- | --- | --- | --- | --- | --- | --- | --- | --- | --- |
| 1023 | *L. lainsoni* | MHOM/BR/1981/M6426 | PA | CL | 15 | 1 | 1 | 2 | 1 | 1 | CC1 |
| 1266 | *L. lainsoni* | MAGO/BR/1983/IM1721 | PA | CL | 15 | 2 | 1 | 2 | 1 | 2 | CC1 |
| 855 | *L. naiffi* | MHOM/BR/1986/IM2773 | AM | * | 36 | 3 | 2 | 1 | 2 | 3 | singl |
| 1365 | *L. naiffi* | MDAS/BR/1979/M5533 | PA | VL | 36 | 4 | 3 | 28 | 3; 4 | 4 | htz |
| 854 | *L. naiffi* | ISQU/BR/1985/IM2264 | PA | * | 38 | 4 | 7 | 6 | 3 | 5 | CC2 |
| 1939 | *L. naiffi* | ISQU/BR/1994/IM3936 | AM | * | 49 | 4 | 7 | 6 | 10 | 6 | CC2 |
| 1953 | *L. naiffi* | MHOM/BR/1994/IM4000 | AM | CL | 49 | 4 | 7 | 7 | 3 | 7 | CC2 |
| 1545 | *L. shawi* | MCEB/BR/1984/M8408 | PA | CL | 26 | 5 | 4 | 3 | 5 | 8 | CC3 |
| 3199 | *L. shawi* | MHOM/BR/1999/M17997 | PA | * | * | 5 | 4 | 3 | 8 | 9 | CC3 |
| 3200 | *L. shawi* | MHOM/BR/1999/M17998 | PA | * | * | 5 | 4 | 3 | 9 | 10 | CC3 |
| 1731 | *L. braziliensis* | MAGO/BR/1992/IM154 | RO | CL | 53 | 6 | 5 | 1 | 6 | 11 | singl |
| 849 | *L. braziliensis* | MHOM/BR/1987/J.CARLOS | RJ | MCL | 27 | 7 | 6 | 1 | 6 | 12 | CC4 |
| 2139 | *L. braziliensis* | MHOM/BR/1996/GBS | PB | CL | 27 | 7 | 6 | 1 | 6 | 12 | CC4 |
| 2159 | *L. braziliensis* | MHOM/BR/1996/JPS | RJ | CL | 27 | 7 | 6 | 1 | 6 | 12 | CC4 |
| 2287 | *L. braziliensis* | MHOM/BR/1998/AFS | PE | CL | 72 | 7 | 6 | 1 | 6 | 12 | CC4 |
| 2288 | *L. braziliensis* | MHOM/BR/1996/MAS | PE | CL | 45 | 7 | 6 | 1 | 6 | 12 | CC4 |
| 2291 | *L. braziliensis* | MHOM/BR/1997/ASB | PE | MCL | 73 | 7 | 6 | 1 | 6 | 12 | CC4 |
| 2419 | *L. braziliensis* | MHOM/BR/1999/JAS | PE | CL | 73 | 7 | 6 | 1 | 6 | 12 | CC4 |
| 2420 | *L. braziliensis* | MHOM/BR/1999/PCS | PE | CL | 45 | 7 | 6 | 1 | 6 | 12 | CC4 |
| 2427 | *L. braziliensis* | MHOM/BR/1999/SJB | PE | CL | 74 | 7 | 6 | 1 | 6 | 12 | CC4 |
| 2467 | *L. braziliensis* | MHOM/BR/2001/LTCP14182 | BA | DL | 27 | 7 | 6 | 1 | 6 | 12 | CC4 |
| 2472 | *L. braziliensis* | MHOM/BR/2001/LTCP13455 | BA | CL | 27 | 7 | 6 | 1 | 6 | 12 | CC4 |
| 2475 | *L. braziliensis* | MHOM/BR/2001/LTCP14214 | BA | CL | 27 | 7 | 6 | 1 | 6 | 12 | CC4 |
| 2480 | *L. braziliensis* | MHOM/BR/2001/LTCP13980 | BA | MCL | 27 | 7 | 6 | 1 | 6 | 12 | CC4 |
| 2510 | *L. braziliensis* | MHOM/BR/2000/CEA | PE | CL | 27 | 7 | 6 | 1 | 6 | 12 | CC4 |
| 2515 | *L. braziliensis* | MHOM/BR/2001/TSS | PE | CL | 78 | 7 | 6 | 1 | 6 | 12 | CC4 |
| 2660 | *L. braziliensis* | MHOM/BR/2004/ARARAQUARA-1 | SP | CL | 27 | 7 | 6 | 1 | 6 | 12 | CC4 |
| 2836 | *L. braziliensis* | MHOM/BR/1994/LTCP9845 | BA | CL | 27 | 7 | 6 | 1 | 6 | 12 | CC4 |
| 2838 | *L. braziliensis* | MHOM/BR/1997/LTCP11245 | BA | CL | 27 | 7 | 6 | 1 | 6 | 12 | CC4 |
| 2148 | *L. braziliensis* | MHOM/BR/2001/VLNC | PE | MCL | 105 | 7 | 6 | 1 | 11 | 13 | CC4 |
| 2481 | *L. braziliensis* | MHOM/BR/2000/LTCP13490 | BA | CL | 27 | 7 | 6 | 1 | 14 | 14 | CC4 |
| 2502 | *L. braziliensis* | MHOM/BR/2002/NMT-RBO040 | AC | CL | 82 | 7 | 6 | 1 | 25 | 15 | CC4 |
| iz 26 | *L. braziliensis* | MORY/PE/84/AO23 | - | * | * | 7 | 6 | 1 | 40; 41 | 16 | htz |
| iz 28 | *L. braziliensis* | MRAT/PE/84/A1 | - | * | * | 7 | 6 | 1 | 40; 41 | 16 | htz |
| iz 31 | *L. braziliensis* | MORY/PE/84/AC20 | - | * | * | 7 | 6 | 1 | 42 | 17 | CC4 |
| iz 27 | *L. braziliensis* | MORY/PE/84/AO8 | - | * | * | 7 | 6 | 1 | 6; 41 | 18 | htz |
| 2491 | *L. braziliensis* | MHOM/BR/2002/NMT-RBO 005 | AC | CL | 78 | 7 | 6 | 8 | 16 | 19 | singl |
| 2535 | *L. braziliensis* | MHOM/BR/2001/HC-JS | ES | CL | 27 | 7 | 6 | 8 | 12; 27 | 20 | htz |
| 2344 | *L. braziliensis* | MHOM/BR/1997/NMT-MAO 229P | AM | CL | * | 7 | 6 | 10 | 6 | 21 | CC4 |
| 2693 | *L. braziliensis* | MNEC/BR/2003/NECTOMYS | PE | CL | 74 | 7 | 6 | 12 | 6 | 22 | CC4 |
| 2495 | *L. braziliensis* | MHOM/BR/2002/NMT-RBO025 | AC | CL | 78 | 7 | 6 | 12 | 19 | 23 | CC4 |
| 2950 | *L. braziliensis* | MHOM/BR/2006/CEN | PE | CL | 75 | 7 | 6 | 17 | 33 | 24 | CC5 |
| 2494 | *L. braziliensis* | MHOM/BR/2002/NMT-RBO 018 | AC | CL | 80 | 7 | 6 | 1; 11 | 18 | 25 | htz |
| 1734 | *L. braziliensis* | MHOM/BR/1991/IM3713 | AM | CL | 35 | 7 | 6 | 4; 5 | 7 | 26 | htz |
| 2509 | *L. braziliensis* | MHOM/BR/2001/CRFN | PE | CL | 74 | 7 | 18 | 1 | 6 | 27 | CC4 |
| 2929 | *L. braziliensis* | MHOM/BR/2005/NMT-LTCP16011-P | BA | CL | 27 | 7 | 18 | 1 | 6 | 27 | CC4 |
| iz 33 | *L. braziliensis* | MORY/PE/84/ABR23 | - | * | * | 7 | 18 | 1 | 6; 41 | 28 | htz |
| 2833 | *L. braziliensis* | MHOM/BR/2001/LTCP14349 | BA | MCL | 27 | 7 | 18 | 1; 8 | 6 | 29 | htz |
| 2951 | *L. braziliensis* | MHOM/BR/2006/CM | PE | CL | 75 | 7 | 19 | 17 | 33 | 30 | CC5 |
| 2511 | *L. braziliensis* | MHOM/BR/2001/JS | PE | CL | 105 | 7 | 11; 16 | 1 | 6 | 31 | htz |
| 2499 | *L. braziliensis* | MHOM/BR/2002/NMT-RBO 035 | AC | CL | 81 | 7 | 12; 13 | 1 | 23 | 32 | htz |
| 2468 | *L. braziliensis* | MHOM/BR/2001/LTCP14183 | BA | MCL | 27 | 7 | 6; 11 | 1 | 6 | 33 | htz |
| iz 18 | *L. guyanensis* | MCHO/BR/80/M6202 | * | * | * | 8 | 4 | 9 | 9 | 34 | CC6 |
| 2466 | *L. braziliensis* | MHOM/BR/2001/LTCP13183 | BA | CL | 27 | 8 | 6 | 1 | 6 | 35 | CC4 |
| 2476 | *L. braziliensis* | MHOM/BR/2001/LTCP14278 | BA | DL | 27 | 8 | 6 | 1 | 6 | 35 | CC4 |
| 2847 | *L. braziliensis* | MHOM/BR/2002/LTCP15476 | BA | CL | 27 | 8 | 6 | 1 | 6 | 35 | CC4 |
| 2152 | *L. braziliensis* | MHOM/BR/1996/AFN | RJ | CL | 27 | 8 | 6 | 8 | 12 | 36 | singl |
| 565 | *L. guyanensis* | MHOM/BR/1975/M4147 | PA | CL | 23 | 9 | 4 | 9 | 9 | 37 | CC6 |
| 2963 | *L. guyanensis* | MHOM/BR/2007/033-MECM | AM | CL | 23 | 9 | 4 | 9 | 13 | 38 | CC6 |
| 2937 | *L. guyanensis* | MHOM/BR/2007/065 | AM | CL | 23 | 9 | 4 | 9 | 30 | 39 | CC6 |
| 2971 | *L. guyanensis* | MHOM/BR/2007/021-R | AM | CL | 23 | 9 | 4 | 9 | 37 | 40 | CC6 |
| 2969 | *L. guyanensis* | MHOM/BR/2007/AC | AM | CL | 23 | 9 | 4 | 26 | 30 | 41 | CC6 |
| 2334 | *L. guyanensis* | MHOM/BR/1997/NMT-MAO 202P | AM | CL | 23 | 9 | 8 | 9 | 9 | 42 | CC6 |
| 2341 | *L. guyanensis* | MHOM/BR/1997/NMT-MAO 223P | AM | CL | 23 | 9 | 9 | 9 | 9 | 43 | CC6 |
| 2350 | *L. guyanensis* | MHOM/BR/1997/NMT-MAO 237P | AM | CL | 23 | 9 | 10 | 9 | 13 | 44 | CC6 |
| 2970 | *L. guyanensis* | MHOM/BR/2007/021-HMB | AM | CL | 23 | 9 | 10 | 9 | 36 | 45 | CC6 |
| 2936 | *L. guyanensis* | MHOM/BR/2007/069 | AM | CL | 23 | 9 | 22 | 9 | 9 | 46 | CC6 |
| 2938 | *L. guyanensis* | MHOM/BR/2007/063 | AM | CL | 23 | 9 | 23 | 19 | 31; 32 | 47 | htz |
| 2956 | *L. guyanensis* | MHOM/BR/2007/011 | AM | CL | 23 | 9 | 24 | 9 | 9 | 48 | CC6 |
| 2957 | *L. guyanensis* | MHOM/BR/2007/014-JIS | AM | CL | 23 | 9 | 25 | 9; 20 | 9 | 49 | htz |
| 2961 | *L. guyanensis* | MHOM/BR/2007/019-WDSN | AM | CL | 23 | 9 | 27 | 22 | 9 | 50 | singl |
| 2962 | *L. guyanensis* | MHOM/BR/2007/031-LOP | AM | CL | 23 | 9 | 28 | 23 | 35 | 51 | singl |
| 2966 | *L. guyanensis* | MHOM/BR/2007/039 | AM | CL | 23 | 9 | 29 | 24; 25 | 9 | 52 | htz |
| iz 34 | *L. guyanensis* | MCHO/BR/80/M6200 | * | * | * | 9 | 4; 33 | 32 | 9 | 53 | htz |
| 2490 | *L. lainsoni/ L. naiffi* | MHOM/BR/2002/NMT-RBO004 | AC | CL | 87 | 11 | 14 | 2 | 15 | 54 | singl |
| 2493 | *L. guyanensis* | MHOM/BR/2002/NMT-RBO 013 | AC | CL | 110 | 12 | 4 | 9 | 17 | 55 | singl |
| 2497 | *L. lainsoni* | MHOM/BR/2002/NMT-RBO 027P | AC | CL | 86 | 13 | 1 | 13 | 20; 21 | 56 | htz |
| 2498 | *L. braziliensis* | MHOM/BR/2002/NMT-RBO 029 | AC | CL | 84 | 14 | 15 | 14 | 22 | 57 | singl |
| 2500 | *L. lainsoni* | MHOM/BR/2002/NMT-RBO036 | AC | CL | 86 | 15 | 17 | 1 | 24 | 58 | singl |
| 2501 | *L. braziliensis* | MHOM/BR/2002/NMT-RBO037 | AC | CL | 79 | 16 | 6 | 15 | 6 | 59 | singl |
| 2503 | *L. lainsoni* | MHOM/BR/2002/NMT-RBO044 | AC | CL | 86 | 17 | 14 | 16 | 26 | 60 | singl |
| 566 | *L. braziliensis* | MHOM/BR/1975/M2903 | PA | CL | 27 | 18 | 6 | 1 | 11 | 61 | CC4 |
| 921 | *L. braziliensis* | MHOM/BR/1987/H-210 | CE | CL | 27 | 18 | 6 | 27 | 11 | 62 | CC4 |
| 918 | *L. braziliensis* | MRAT/BR/1987/C18.454 | CE | VL | 27 | 18 | 6 | 8; 11 | 11 | 63 | htz |
| 2538 | *L. braziliensis* | MHOM/BR/2002/EMM | RJ | CL | 27 | 18 | 19 | 8; 17 | 6; 11 | 64 | htz |
| 2541 | *L. braziliensis* | MHOM/BR/1999/MJAA-II | PE | CL | 74 | 19 | 6; 16 | 1 | 6 | 65 | htz |
| 2689 | *L. utingensis* | ITUB/BR/1977/M4964 | PA | * | 101 | 20 | 20 | 18 | 28 | 66 | singl |
| 2690 | *L. lindenbergi* | MHOM/BR/1966/M15733 | PA | CL | 102 | 21 | 21 | 1 | 29 | 67 | singl |
| 2960 | *L. guyanensis* | MHOM/BR/2007/029-ZAV | AM | CL | 23 | 22 | 26 | 21 | 34 | 68 | singl |
| 2964 | *L. guyanensis* | MHOM/BR/2007/034-MFPS | AM | CL | 23 | 23 | 4 | 9 | 9 | 69 | singl |
| 992 | *L. naiffi* | MDAS/BR/1987/IM3280 | PA | VL | 42 | 24 | 30 | 28 | 3 | 70 | singl |
| 995 | *L. naiffi* | MDAS/BR/1987/IM3292 | PA | VL | 36 | 24 | 30; 31 | 29 | 38 | 71 | htz |
| iz 35 | *L. lainsoni* | IUBI/BR/00/M12025 | PA | * | * | 25 | 1 | 33 | 43 | 72 | singl |
| iz 25 | *L. lainsoni* | MHOM/BR/82/M6887 | PA | * | * | 25 | 1; 32 | 30; 31 | 39 | 73 | htz |
| 2463 | *L. braziliensis* | MHOM/BR/2001/JOLIVAL | BA | MCL | 27 | 7; 10 | 6; 11 | 1 | 6 | 74 | htz |
| 2571 | *L. braziliensis* | MHOM/BR/2003/NJS | MS | CL | 7 | 7; 8 | 6 | 1 | 6 | 75 | htz |

From the left: IOC/L (Institute Oswaldo Cruz Leishmania Collection) – culture collection code; *Leishmania* species; International code – see WHO, 2010 for description, BR= Brazil and PE= Peru; Brazilian states – AC: Acre, AM: Amazonas, BA: Bahia, CE: Ceará, PA: Pará, PB: Paraíba, PE: Pernambuco, ES: Espírito Santo, MS: Mato Grosso do Sul, RJ: Rio de Janeiro, RO: Rondônia, SP: São Paulo; clinical form observed in the host: VL – visceral leishmaniasis, CL – cutaneous leishmaniasis, MCL – mucocutaneous leishmaniasis; zymodemes according to the CLIOC system ; sequence types observed for each marker; DST: diploid sequence type; CC: clonal complexes formed after e-burst analysis; singl: singletons after e-burst analysis; htz: heterozygous strains – not included in the e- burst analysis. * Information not available.
