# Supplementary material for: New Insights on Taxonomy, Phylogeny and Population Genetics of Leishmania (Viannia) Parasites Based on Multilocus Sequence Analysis
Source: PLoS Negl Trop Dis. 2012 Nov 1;6(11):e1888. doi: 10.1371/journal.pntd.0001888 (PMC3486886; doi:10.1371/journal.pntd.0001888)
Supplement: Table S2 — Sequences retrieved from GenBank and those included in the analysis performed in the present study, indicating the assigned sequence type (ST) for each marker. (DOCX) [file pntd.0001888.s002.docx]

**Table S2. Sequences retrieved from GenBank and those included in the analysis performed in the present study, indicating the assigned sequence type (ST) for each marker.**

| **Marker** | **GenBank available sequences included in the ST alignments** | **Full sequence cover?*** | **ST** |
| --- | --- | --- | --- |
| **6PGD** | *Leishmania braziliensis* MHOM/BR/75/M2904 6-phosphogluconate dehydrogenasedecarboxylating (LbrM34_V2.3250) | yes | 1 |
|  | *Leishmania peruviana* strain MHOM/PE/87/PAB2941 6-phosphogluconate dehydrogenase (6pgd) gene complete cds | yes | 1 |
|  | *Leishmania peruviana* strain MHOM/PE/92/LEH1563 6-phosphogluconate dehydrogenase (6pgd) gene complete cds | yes | 1 |
|  | *Leishmania peruviana* strain MHOM/PE/92/LEH1384 6-phosphogluconate dehydrogenase (6pgd) gene complete cds | yes | 1 |
|  | *Leishmania peruviana* strain MHOM/PE/92/LEH1339 6-phosphogluconate dehydrogenase (6pgd) gene complete cds | yes | 1 |
|  | *Leishmania peruviana* strain MHOM/PE/91/LEH0521 6-phosphogluconate dehydrogenase (6pgd) gene complete cds | yes | 1 |
|  | *Leishmania peruviana* strain MHOM/PE/87/PAB2885 6-phosphogluconate dehydrogenase (6pgd) gene complete cds | yes | 1 |
|  | *Leishmania peruviana* strain MHOM/PE/92/LEH1449 6-phosphogluconate dehydrogenase (6pgd) gene complete cds | yes | 1 |
|  | *Leishmania peruviana* strain MHOM/PE/92/LEH1347 6-phosphogluconate dehydrogenase (6pgd) gene complete cds | yes | 1 |
|  | *Leishmania peruviana* strain MHOM/PE/91/LEH0547 6-phosphogluconate dehydrogenase (6pgd) gene complete cds | yes | 1 |
|  | *Leishmania peruviana* strain MHOM/PE/87/PAB2880 6-phosphogluconate dehydrogenase (6PGD) gene complete cds | yes | 1 |
|  | *Leishmania braziliensis* strain MHOM/PE/93/LEH1642 6-phosphogluconate dehydrogenase (6pgd) gene complete cds | yes | 15 |
|  | *Leishmania braziliensis* strain MHOM/PE/94/LEH1829 6-phosphogluconate dehydrogenase (6pgd) gene complete cds | yes | 15 |
|  | *Leishmania braziliensis* strain MHOM/PE/91/LEH0375 6-phosphogluconate dehydrogenase (6pgd) gene complete cds | yes | 15 |
|  | *Leishmania braziliensis* strain MHOM/PE/93/LEH0022 6-phosphogluconate dehydrogenase (6pgd) gene complete cds | yes | 15 |
|  | *Leishmania braziliensis* strain MHOM/PE/88/BAA2079 6-phosphogluconate dehydrogenase (6PGD) gene complete cds | yes | New-35 |
|  | *Leishmania braziliensis* strain MHOM/BR/84/LTB300 6-phosphogluconate dehydrogenase (6PGD) gene complete cds | yes | 1 |
|  | *Leishmania panamensis* strain MHOM/PA/71/LS94 6-phosphogluconate dehydrogenase (6PGD) gene complete cds | yes | New-36 |
|  | *Leishmania guyanensis* strain MHOM/BR/75/M4147 6-phosphogluconate dehydrogenase (6PGD) gene complete cds | no | .. |
|  | *Leishmania lainsoni* strain MHOM/PE/88/BAB1730 6-phosphogluconate dehydrogenase (6PGD) gene complete cds | no | .. |
|  | *Leishmania braziliensis* 6-phosphogluconate dehydrogenase gene partial cds | no | .. |
|  | *Leishmania guyanensis* 6-phosphogluconate dehydrogenase gene partial cds | no | .. |
|  | *Leishmania panamensis* 6-phosphogluconate dehydrogenase gene partial cds | no | .. |
|  | *Leishmania lainsoni* 6-phosphogluconate dehydrogenase gene partial cds | no | .. |
| **G6PD** | *Leishmania braziliensis* strain MHOM/BR/1975/M2903 glucose-6-phosphate dehydrogenase (G6PD) mRNA partial cds | no | ... |
|  | *Leishmania peruviana* strain MHOM/PE/1984/LC39 glucose-6-phosphate dehydrogenase (G6PD) mRNA partial cds | no | ... |
|  | *Leishmania guyanensis* strain MHOM/BR/1975/M4147 glucose-6-phosphate dehydrogenase (G6PD) mRNA complete cds | no | ... |
|  | *Leishmania panamensis* strain MHOM/PA/1971/LS94 glucose-6-phosphate dehydrogenase (G6PD) mRNA partial cds | no | ... |
|  | *Leishmania lainsoni* strain MHOM/BR/1981/M6426 glucose-6-phosphate dehydrogenase (G6PD) mRNA partial cds | no | ... |
|  | *Leishmania braziliensis* strain MHOM/BR/84/LTB300 glucose-6-phosphate dehydrogenase (G6PD) gene complete cds | yes | 6 |
|  | *Leishmania braziliensis* strain MHOM/PE/88/BAA2079 glucose-6-phosphate dehydrogenase (G6PD) gene complete cds | yes | New-34 |
|  | *Leishmania braziliensis* strain MHOM/PE/88/BAA2072 glucose-6-phosphate dehydrogenase (G6PD) gene complete cds | yes | 6 |
|  | *Leishmania braziliensis* strain MDID/BR/1995/IM4159 glucose-6-phosphate dehydrogenase (G6PD) mRNA partial cds | no | ... |
|  | *Leishmania braziliensis* strain MAGO/BR/1992/IM154 glucose-6-phosphate dehydrogenase (G6PD) mRNA partial cds | no | ... |
|  | *Leishmania peruviana* strain MHOM/PE/87/PAB2880 glucose-6-phosphate dehydrogenase (G6PD) gene complete cds | yes | 6 |
|  | *Leishmania guyanensis* strain MHOM/BR/75/M4147 glucose-6-phosphate dehydrogenase (G6PD) gene complete cds | yes | 4 |
|  | *Leishmania panamensis* strain MHOM/PA/71/LS94 glucose-6-phosphate dehydrogenase (G6PD) gene complete cds | yes | New-36 |
|  | *Leishmania lainsoni* strain MHOM/PE/88/BAB1730 glucose-6-phosphate dehydrogenase (G6PD) gene complete cds | yes | New-37 |
|  | *Leishmania braziliensis* MHOM/BR/75/M2904_glucose-6-phosphate_dehydrogenase_(LbrM20_V2.0160)_partial_mRNA | yes | New-35 |
| **ICD** | *Leishmania braziliensis* MHOM/BR/75/M2904 isocitrate dehydrogenase putative (LbrM33_V2.2820) partial mRNA | yes | 6 |
| **MPI** | *Leishmania peruviana* strain MHOM/PE/87/PAB2941 mannose phosphate isomerase (mpi) gene complete cds | yes | New-7;26** |
|  | *Leishmania peruviana* strain MHOM/PE/92/LEH1339 mannose phosphate isomerase (mpi) gene complete cds | yes | 7 |
|  | *Leishmania peruviana* strain MHOM/PE/92/LEH1384 mannose phosphate isomerase (mpi) gene complete cds | yes | 7 |
|  | *Leishmania peruviana* strain MHOM/PE/92/LEH1449 mannose phosphate isomerase (mpi) gene complete cds | yes | 7 |
|  | *Leishmania peruviana* strain MHOM/PE/91/LEH0521 mannose phosphate isomerase (mpi) gene complete cds | yes | New-27 |
|  | *Leishmania peruviana* strain MHOM/PE/92/LEH1563 mannose phosphate isomerase (mpi) gene complete cds | yes | 7 |
|  | *Leishmania peruviana* strain MHOM/PE/92/LEH1347 mannose phosphate isomerase (mpi) gene complete cds | yes | 7 |
|  | *Leishmania peruviana* strain MHOM/PE/87/PAB2885 mannose phosphate isomerase (mpi) gene complete cds | yes | New-7;26** |
|  | *Leishmania peruviana* strain MHOM/PE/91/LEH0547 mannose phosphate isomerase (mpi) gene complete cds | yes | 7 |
|  | *Leishmania peruviana* strain MHOM/PE/87/PAB2880 mannose phosphate isomerase (MPI) gene complete cds | yes | 7 |
|  | *Leishmania peruviana* strain MHOM/PE/90/HB44 mannose phosphate isomerase gene complete cds | yes | 7 |
|  | *Leishmania braziliensis* strain MHOM/PE/93/LEH1642 mannose phosphate isomerase (mpi) gene complete cds | yes | 7 |
|  | *Leishmania braziliensis* strain MHOM/PE/94/LEH1829 mannose phosphate isomerase (mpi) gene complete cds | yes | 7 |
|  | *Leishmania braziliensis* strain MHOM/PE/93/LEH0022 mannose phosphate isomerase (mpi) gene complete cds | yes | 7 |
|  | *Leishmania braziliensis* strain MHOM/PE/91/LEH0375 mannose phosphate isomerase (mpi) gene complete cds | yes | 7 |
|  | *Leishmania braziliensis* strain MHOM/BR/84/LTB300 mannose phosphate isomerase (MPI) gene complete cds | yes | 7 |
|  | *Leishmania braziliensis* strain MHOM/PE/88/BAA2079 mannose phosphate isomerase (MPI) gene complete cds | yes | New-28 |
|  | *Leishmania braziliensis* strain MHOM/PE/88/BAA2072 mannose phosphate isomerase (MPI) gene complete cds | yes | 7 |
|  | *Leishmania braziliensis* MHOM/BR/75/M2904 phosphomannose isomerase putative (LbrM32_V2.1750) partial mRNA | yes | 7 |
|  | *Leishmania braziliensis* strain MHOM/BR/91/2195 mannose phosphate isomerase gene complete cds | yes | 7 |

Available sequences for each marker included in the Sequence Types (STs) alignment for comparison regarding coverage* – which allows comparison or not – and ST found for the respective strain. ** A new heterozygous sequence type.
