# Supplementary material for: New Insights on Taxonomy, Phylogeny and Population Genetics of Leishmania (Viannia) Parasites Based on Multilocus Sequence Analysis
Source: PLoS Negl Trop Dis. 2012 Nov 1;6(11):e1888. doi: 10.1371/journal.pntd.0001888 (PMC3486886; doi:10.1371/journal.pntd.0001888)
Supplement: Table S3 — Multi-alleles sites observed for each fragment-gene alignment and the distribution of each allele in the studied strains. (DOCX) [file pntd.0001888.s003.docx]

**Table S3. Multi-alleles sites observed for each fragment-gene alignment and the distribution of each allele in the studied strains.**

| **Marker** | **Position** | **Nucleotide** | **Number of strains presenting the nucleotide / total number of strains** |
| --- | --- | --- | --- |
| G6PD | 179 | A | 5/7 *L. naiffi* |
|  |  | C | 21/22 *L. guyanensis*; 3/*3 L. shawi*; 1/1 *L. utingensis* |
|  |  | G | All other strains |
|  | 341 | C | 7/7 *L. naiffi* |
|  |  | A | 21/22 *L. guyanensis*; 3/3 *L. shawi* |
|  |  | G | All other strains |
|  | 467 | T | 1/55 *L. braziliensis* |
|  |  | A | 7/7 *L. naiffi* |
|  |  | C | All other strains |
| 6PGD | 9 | T | 3/7 *L. naiffi*; 2/55 *L. braziliensis* |
|  |  | G | 1/22 *L. guyanensis* |
|  |  | C | All other strains |
|  | 90 | A | 3/8 *L. lainsoni*; 2/22 *L. guyanensis* |
|  |  | C | 3/7 *L. naiffi*; 1/8 *L. lainsoni*; 2/55 *L. braziliensis* |
|  |  | T | All other strains |
| ICD | 52 | C | 1/55 *L. braziliensis* |
|  |  | T | 1/3 *L. shawi* |
|  |  | G | All other strains |
|  | 97 | T | 1/1 *L. utingensis*; 1/55 *L. braziliensis* |
|  |  | C | 1/22 *L. guyanensis* |
|  |  | G | All other strains |
