# Supplementary material for: New Insights on Taxonomy, Phylogeny and Population Genetics of Leishmania (Viannia) Parasites Based on Multilocus Sequence Analysis
Source: PLoS Negl Trop Dis. 2012 Nov 1;6(11):e1888. doi: 10.1371/journal.pntd.0001888 (PMC3486886; doi:10.1371/journal.pntd.0001888)
Supplement: Table S4 — Strains presenting ambiguous sites (IUPAC symbols) for the targets with the respective site position in length alignment and the most common nucleotide. (DOCX) [file pntd.0001888.s004.docx]

**Table S4. Strains presenting ambiguous sites (IUPAC symbols) for the targets with the respective site position in length alignment and the most common nucleotide.**

|  |  | **G6PD** | | | | | | **6PGD** | | | | | **MPI** | | | **ICD** | | | | | |
| --- | --- | --- | --- | --- | --- | --- | --- | --- | --- | --- | --- | --- | --- | --- | --- | --- | --- | --- | --- | --- | --- |
| **IOC/L** | ***Species*** | **228** | **316** | **416** | **524** | **538** | **557** | **391** | **460** | **466** | **562** | **602** | **259** | **319** | **480** | **147** | **174** | **318** | **384** | **401** | **492** |
|  | Nucleotides* | G | G | G | C | C | G | C/G/T | T/C | C/T | C | A | A | C/T | A | A/C | C/T | C | G/A | T | T/C |
| 918 | *L. braziliensis* | ... | ... | ... | ... | ... | ... | ... | Y | ... | ... | ... | ... | ... | ... | ... | ... | ... | ... | ... | ... |
| 1734 | *L. braziliensis* | ... | ... | ... | ... | ... | ... | S | ... | ... | ... | ... | ... | ... | ... | ... | ... | ... | ... | ... | ... |
| 2463 | *L. braziliensis* | ... | ... | ... | Y | ... | ... | ... | ... | ... | ... | ... | ... | ... | ... | ... | ... | ... | ... | ... | ... |
| 2468 | *L. braziliensis* | ... | ... | ... | Y | ... | ... | ... | ... | ... | ... | ... | ... | ... | ... | ... | ... | ... | ... | ... | ... |
| 2494 | *L. braziliensis* | ... | ... | ... | ... | ... | ... | S | ... | ... | ... | ... | ... | ... | ... | ... | ... | ... | ... | ... | ... |
| 2499 | *L. braziliensis* | ... | ... | ... | Y | Y | ... | ... | ... | ... | ... | ... | ... | ... | ... | ... | ... | ... | ... | ... | ... |
| 2511 | *L. braziliensis* | ... | ... | ... | ... | ... | ... | ... | ... | ... | ... | ... | W | .... | .... | ... | ... | ... | ... | ... | ... |
| 2535 | *L. braziliensis* | ... | ... | ... | ... | ... | ... | ... | ... | ... | ... | ... | ... | ... | ... | ... | Y | ... | ... | ... | ... |
| 2538 | *L. braziliensis* | ... | ... | ... | ... | ... | ... | S | Y | Y | ... | ... | ... | ... | ... | M | Y | ... | ... | ... | ... |
| 2541 | *L. braziliensis* | ... | ... | ... | ... | Y | ... | ... | ... | ... | ... | ... | ... | ... | ... | ... | ... | ... | ... | ... | ... |
| 2571 | *L. braziliensis* | ... | ... | ... | ... | ... | ... | ... | ... | ... | ... | ... | ... | Y | ... | ... | ... | ... | ... | ... | ... |
| 2833 | *L. braziliensis* | ... | ... | ... | ... | ... | ... | ... | Y | ... | ... | ... | ... | ... | ... | ... | ... | ... | ... | ... | ... |
| iz 26 | *L. braziliensis* | ... | ... | ... | ... | ... | ... | ... | ... | ... | ... | ... | ... | ... | ... | M | Y | ... | ... | ... | ... |
| iz 27 | *L. braziliensis* | ... | ... | ... | Y | ... | ... | ... | ... | ... | ... | ... | ... | ... | ... | M | Y | ... | ... | ... | Y |
| iz 28 | *L. braziliensis* | ... | ... | ... | ... | ... | ... | ... | ... | ... | ... | ... | ... | ... | ... | M | Y | ... | ... | ... | ... |
| iz 31 | *L. braziliensis* | ... | ... | ... | ... | ... | ... | ... | ... | ... | ... | ... | ... | ... | ... | M | ... | ... | ... | ... | ... |
| iz 33 | *L. braziliensis* | ... | ... | ... | ... | ... | ... | ... | ... | ... | ... | ... | ... | ... | ... | M | Y | ... | ... | ... | Y |
| 2938 | *L. guyanensis* | ... | ... | ... | ... | ... | ... | ... | ... | ... | ... | ... | ... | ... | ... | ... | ... | ... | ... | Y | ... |
| iz 34 | *L. guyanensis* | R | ... | R | ... | ... | ... | ... | ... | ... | ... | ... | ... | ... | ... | ... | ... | ... | ... | ... | ... |
| 2957 | *L. guyanensis* | ... | ... | ... | ... | ... | ... | ... | ... | ... | ... | R | ... | ... | ... | ... | ... | ... | ... | ... | ... |
| 2966 | *L. guyanensis* | ... | ... | ... | ... | ... | ... | ... | ... | ... | ... | R | ... | ... | ... | ... | ... | ... | ... | ... | ... |
| 2497 | *L. lainsoni* | ... | ... | ... | ... | ... | ... | ... | ... | ... | ... | ... | ... | ... | ... | ... | ... | ... | Y | ... | ... |
| iz 25 | *L. lainsoni* | ... | ... | ... | ... | ... | R | ... | ... | ... | Y | ... | ... | ... | ... | ... | ... | ... | ... | ... | ... |
| 854 | *L. naiffi* | ... | ... | ... | ... | ... | ... | S | Y | ... | ... | ... | ... | ... | ... | ... | ... | ... | ... | ... | ... |
| 995 | *L. naiffi* | ... | R | ... | ... | ... | ... | ... | ... | ... | ... | ... | ... | ... | ... | ... | ... | ... | ... | ... | ... |
| 1365 | *L. naiffi* | ... | ... | ... | ... | ... | ... | ... | ... | ... | ... | ... | ... | ... | ... | ... | ... | Y | ... | ... | ... |
| .... | ***L. peruviana* | ... | ... | ... | ... | ... | ... | ... | ... | ... | ... | ... | …. | …. | Y | ... | ... | ... | ... | ... | ... |

*Nucleotides: possible nucleotides observed in the alignment, presented accordingly to their frequencies, most frequent to less frequent observed. **Sequence retrieved from GenBank for MPI.
